# Possible Eye Disorders in Children Prenatally Exposed to Either Methadone or Buprenorphine in Comparison with Other Medications: An Examination of the Food and Drug Administration (FDA) Pharmacovigilance Database

**DOI:** 10.3390/brainsci15111177

**Published:** 2025-10-30

**Authors:** Stefania Chiappini, Laura Orsolini, John Martin Corkery, Amira Guirguis, Alessio Mosca, Davide Arillotta, Giovanni Martinotti, Fabrizio Schifano

**Affiliations:** 1 School of Medicine, UniCamillus University, Via di S. Alessandro 8, 00131 Rome, Italy; 2 Unit of Clinical Psychiatry, Department of Experimental and Clinical Medicine (DIMSC), Faculty of Medicine and Surgery, Polytechnic University of Marche, 60126 Ancona, Italy; l.orsolini@staff.univpm.it; 3 Psychopharmacology, Drug Misuse and Novel Psychoactive Substances Research Unit, Hertfordshire Medical School, University of Hertfordshire, Hatfield AL10 9AB, UK; j.corkery@herts.ac.uk (J.M.C.); f.schifano@herts.ac.uk (F.S.); 4 Pharmacy, Medical School, Swansea University, Swansea SA2 8PP, UK; amira.guirguis@swansea.ac.uk; 5 Department of Neuroscience, Imaging and Clinical Sciences, “G. D’Annunzio” University, 66100 Chieti, Italy; alessio.mosca909@gmail.com (A.M.); giovanni.martinotti@gmail.com (G.M.); 6 Department of Neurosciences, Psychology, Drug Research, and Child Health, Section of Pharmacology and Toxicology, University of Florence, 50121 Florence, Italy; davide.arillotta@yahoo.it

**Keywords:** eye disorders, opioids, pharmacovigilance, methadone, buprenorphine, FAERS

## Abstract

**Background/Objectives**: Recent studies have identified some concerns related to the occurrence of eye disorders in offspring of opioid-prescribed mothers, and especially so in those exposed to methadone. The aim here was to investigate, from a pharmacovigilance point of view, the association between opioid exposure during pregnancy and reported eye disorders in children. **Methods**: The FDA Adverse Event Reporting System (FAERS) was searched for the following: reports of eye disorders in children aged 0–17 years exposed during pregnancy to either methadone or buprenorphine; top 20 medications administered during pregnancy and associated with eventual occurrence of eye disorders in children; and reports of eye disorders in children from mothers prescribed with a range of psychotropics. **Results**: For 190 methadone and 79 buprenorphine cases, occurrence of eye disorders was registered as the consequence of having been exposed to these drugs in utero. After data cleaning, residual cases for methadone and buprenorphine were 17 and 15, respectively. Overall, in comparing the odds of eye disorders given methadone exposure to the odds of eye disorders given buprenorphine exposure, which represents a relative Reporting Odds Ratio (ROR) between two drugs, the relative ROR between methadone and buprenorphine was 0.59, suggesting lower odds of eye disorders for methadone compared to buprenorphine in children 0–17 years old antenatally exposed to either methadone or buprenorphine. Conversely, the ROR values resulting from a comparison of methadone- or buprenorphine-related data versus all other psychotropic drugs resulted in 0.27 (95% CI 0.16–0.48) and 0.47 (95% CI 0.26–0.85), respectively, indicating lower reporting odds of eye disorders for these molecules versus the pooled non-opioid comparator group. Medications prescribed during pregnancy which were most frequently related to the occurrence of eye disorders included the following: dupilumab (126 reports), valproate (69 reports), and ibuprofen (52 reports). Indeed, no opiates/opioids appeared among the top 20 drugs linked to eye disorders. A total of 25 and 11 unique cases were associated either with benzodiazepines or antipsychotics, respectively. **Conclusions**: No potential disproportionality safety signal for eye disorders associated with prenatal opioid exposure was identified. Specifically, the relative ROR indicated lower reporting odds for methadone compared to buprenorphine. The interpretation of these results is complicated by common co-exposures, polydrug interventions, and underlying maternal comorbidities, which introduce substantial confounding in real-world pharmacovigilance data. Overall, these findings highlight the importance of continued systematic post-marketing surveillance.

## 1. Introduction

Drug safety during pregnancy remains a critical concern in clinical practice and public health. Maternal exposure to pharmacological agents can have profound consequences on foetal development, including the risk of congenital anomalies and long-term health outcomes in exposed children [1,2]. Opioids are of particular interest in this context due to their widespread use for both acute and chronic pain management, as well as for opioid use disorder treatment in pregnancy.

To date, methadone and buprenorphine are considered as recommended medications for pregnant women with opioid use disorder [3]. According to the American College of Obstetricians and Gynaecologists (ACOG), opioid agonist pharmacotherapy is preferable to medically supervised withdrawal, being opioid withdrawal associated with high relapse rates and worse outcomes [4]. Buprenorphine, its active metabolite norbuprenorphine, and methadone cross the placenta, resulting in neonatal concentrations that can reach approximately 50%, 65%, and 60% of maternal levels, respectively [5,6]. For this reason, their use needs to be weighted according to their risks and benefits profile. Methadone and buprenorphine administration can be continued regardless of the pregnancy term; moreover, due to pharmacokinetic reasons, dosage adjustments (e.g., increasing the dose) may be necessary to maintain their effectiveness [5,6]. At the same time, prolonged use of opioids throughout pregnancy, methadone and buprenorphine included, may result in neonatal withdrawal [7,8], also known as neonatal abstinence syndrome (NAS) or neonatal opioid withdrawal syndrome (NOWS), which may itself temporarily impact some visual functions. Therefore, in case of opioid substitution, the lowest effective dose should be given for safety reasons, especially near term. Whilst their teratogenic potential has been investigated with respect to neural tube defects, congenital heart disease, and neonatal abstinence syndrome [9,10], the relationship between maternal opioid exposure and eye disorders in offspring remains poorly characterised and mostly associated with case reports, case series or research with small numbers of patients [11,12,13,14]. The limited attention to this area has been attributed to several factors: low rates of attendance of drug-using mothers at paediatric ophthalmology appointments, reluctance to engage with services associated with prior addiction, and concerns about disclosing a history of opioid use to current partners [13]. Additional methodological challenges arise from the high prevalence of polydrug exposure in this population, which complicates causal attribution, and from stigma-related underreporting of substance use [13]. Despite these limitations, there are compelling reasons to suspect that visual functioning in children with prenatal opioid exposure may be compromised. Evidence from electrophysiological studies, particularly those measuring visual evoked potentials (VEPs), indicates altered visual pathway processing in this group of children exposed in utero to opiates/opioids [15,16,17,18]. This knowledge gap is clinically significant given the global prevalence of opioid use and the potential impact of early visual impairment on neurodevelopment. Pharmacovigilance databases, such as the FDA Adverse Event Reporting System (FAERS), provide a valuable resource to explore rare or unexpected safety signals in large populations [19,20]. Although these voluntary reporting systems are limited by underreporting and variable data quality, they allow the identification of associations between drug exposure and adverse events that may not be captured in clinical trials, e.g., the possible misuse of medications such as the over-the-counter anti-diarrhoeal loperamide [21] or the antiepileptic drug pregabalin [22], then leading to specific strategies and interventions to reduce the possible related risks in special categories of patients [23,24]. Analysing pharmacovigilance data specifically in paediatric populations can shed light on drug-related risks during early life stages, including those linked to in utero exposure.

The aim of this study was the following: the present study aimed to investigate the association between maternal opioid exposure during pregnancy and reported eye disorders in children, including congenital eye disorders. By systematically examining pharmacovigilance data from FAERS, we sought to identify the most frequently reported drugs associated with these outcomes and to place opioids in a context relating to other pharmacological classes of interest, such as benzodiazepines, anticholinergic agents, antipsychotics, and gabapentinoids.

## 2. Materials and Methods

A retrospective analysis was conducted using data from the FAERS, a publicly accessible pharmacovigilance database (https://www.fda.gov/drugs/fdas-adverse-event-reporting-system-faers/fda-adverse-event-reporting-system-faers-public-dashboard, accessed on 11 September 2025). The database was searched for reports of ‘eye disorders’ in children aged 0–17 years exposed during pregnancy to a specific drug, e.g., methadone and buprenorphine (up to 2025); for completeness, the same search was carried out with a range of prescribed psychotropics, including benzodiazepines, antipsychotics, anticholinergics, and gabapentinoids. To ensure data accuracy and prevent overestimation, duplicate reports were identified and removed based on matching case IDs and overlapping clinical information. Descriptive statistics were applied to summarise the distribution of eye disorder reports across age groups, drug classes, and individual substances. Cases were summarised and, for each one, reported relevant clinical information, including eye-related Adverse Drug Reports (ADRs), other ADRs, concomitant drugs/medications, reporter, year of the report, and age and sex of the patient; references from the literature were collated. ROR values resulting from a comparison of methadone- and buprenorphine-related data and methadone or buprenorphine versus all other psychotropic drugs were computed. In a subsequent analysis, to better understand the association between opioid use during pregnancy and the potential occurrence of visual disorders in offspring, the entire FAERS dataset (up to 2025) was queried for ADRs coded under the Preferred Terms (PTs) ‘eye disorders’ and ‘congenital eye disorders’ in the paediatric population (age 0–17 years). In addition to the first one, this analysis aimed to identify the most frequently reported drugs, distinguishing between reports of general eye disorders and congenital eye defects. This approach was chosen to allow a comparison of opioids with other pharmacological classes whilst possibly providing insight into patterns of drug-associated ocular adverse events in children. Thirdly, in addition to opioids, other pharmacological categories of interest, e.g., benzodiazepines (e.g., diazepam, alprazolam, clonazepam, lorazepam, and bromazepam), anticholinergic drugs (e.g., biperiden, benztropine, trihexyphenidyl); gabapentinoids (e.g., pregabalin and gabapentin), and antipsychotics (e.g., quetiapine, olanzapine, and clozapine), were analysed in a similar manner.

## 3. Results

### 3.1. Methadone and Buprenorphine-Related Eye Disorders Cases Recorded in 0–17-Year-Old Individuals

From the FAERS database (up to 1 September 2025), the number of ‘eye disorders’ reported in children (0–17 years) exposed to opioids such as methadone and buprenorphine (where buprenorphine including the combo buprenorphine/naloxone) during pregnancy were identified. 

The total number of ‘eye disorders’ and ADRs involving subjects aged 0–17 years old was 2297; for 190 of these cases, the ADR was registered as the consequence of having been exposed to *methadone* in utero. However, some cases recorded with specific PTs had to be excluded due to a range of reasons, including accidental exposure to methadone (37 cases) and duplicates (113 cases). The characteristics of the remaining 17 cases are described in Table 1. Conversely, the total number of ‘eye disorders’, buprenorphine-related ADRs involving subjects aged 0–17 years old, was 1199; for 79 of these cases, the ADR was noted as being the consequence of having been exposed to *either buprenorphine* or *buprenorphine/naloxone* in utero. The following cases had to be excluded due to a range of reasons, including accidental poisoning (22 cases) and duplicates (36 cases). The remaining 15 cases are described in detail in Table 1; for a detailed description of cases, whether related to methadone or buprenorphine, please refer to Supplementary Tables S1–S3.

**Figure 1:**
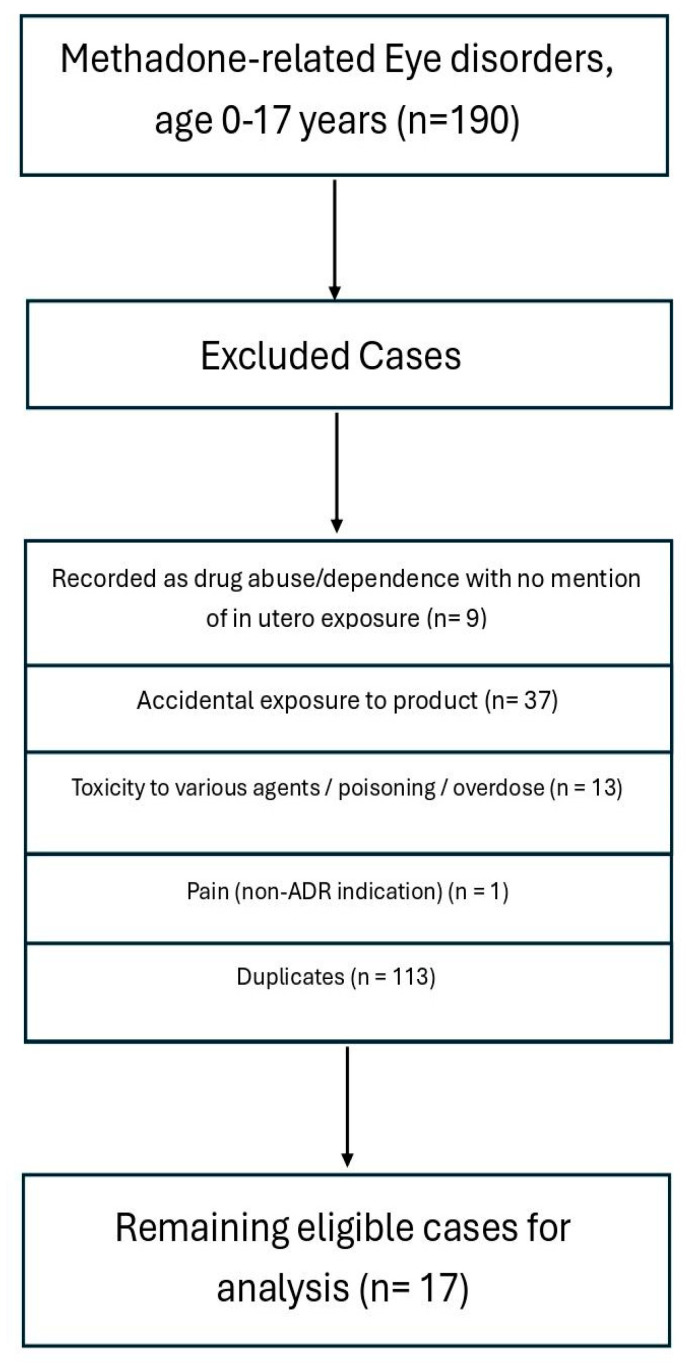
Case selection flow chart for methadone cases.

**Figure 2:**
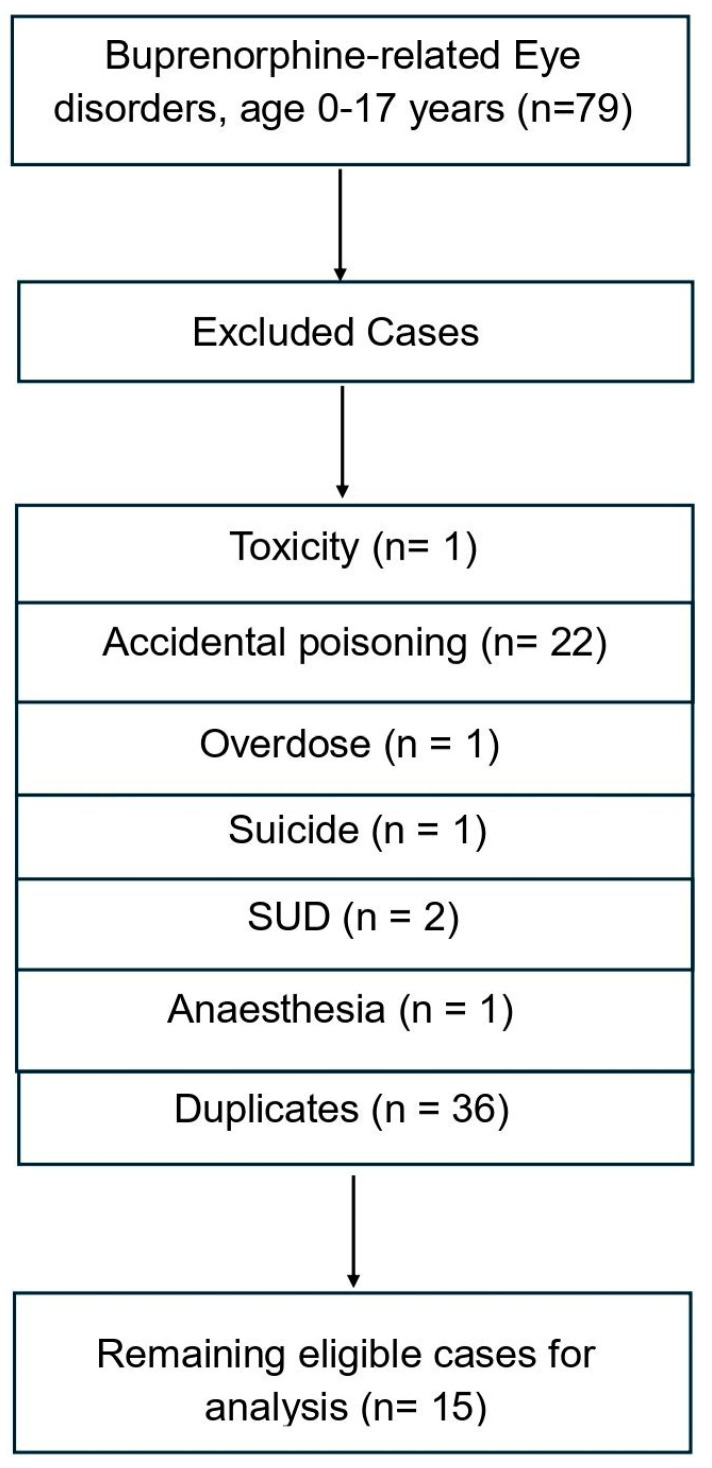
Case selection flow chart for buprenorphine cases.

The case selection process for eye-related methadone and buprenorphine ADRs among individuals aged 0–17 years is illustrated in Figure 1 and Figure 2 and described in detail here. In the first case, a total of 190 reports were initially identified. Cases were excluded if, according to the PTs, they were recorded as drug abuse or dependence without mention of in utero exposure (*n* = 9), accidental exposure to the product (*n* = 37), toxicity or poisoning due to various agents or overdose (*n* = 13); non-ADR indications such as pain (*n* = 1) were identified as duplicate entries (*n* = 113). After applying these exclusion criteria, 17 unique and relevant cases remained for inclusion in the final analysis. Similarly, in the case of buprenorphine, the selection process for buprenorphine-related eye disorder ADRs among individuals aged 0–17 years was reported in a flow chart. A total of 79 reports were initially identified. Cases were excluded if classified as toxicity (*n* = 1), accidental poisoning (*n* = 22), overdose (*n* = 1), suicide (*n* = 1), substance use disorder (SUD; *n* = 2), anaesthesia-related (*n* = 1), or duplicate reports (*n* = 36). After applying these exclusion criteria, 15 unique cases remained for inclusion in the final analysis.

Overall, reported ocular adverse events included nystagmus, strabismus, amblyopia, reduced visual acuity, optic nerve hypoplasia, retinopathy, and visual impairment, often in association with developmental delays, congenital anomalies, neonatal withdrawal syndrome, or low birth weight. Some of the reports were supported by published case series or observational studies, describing ophthalmic abnormalities in children exposed in utero to opioids (see Supplementary Table S1).

Considering the above, the ROR values resulting from a comparison of methadone- or buprenorphine-related data were calculated as follows:

- a = methadone associated with eye disorders = 17;
- b = methadone without eye disorders = 2297 − 17 = 2280;
- c = buprenorphine with eye disorders = 15;
- d = buprenorphine without eye disorders = 1199 − 15 = 1184.

With ROR being the following: (a/b)/(c/d), ROR = 0.59 (95% CI = 0.29–1.18; n.s.). This ROR compared the odds of eye disorders given methadone exposure to the odds of eye disorders given buprenorphine exposure. This is a *relative ROR between two drugs.* If interpreting ROR > 1 as a signal and ROR < 1 as protective, the ROR of 0.59 may well suggest that the odds of eye disorders are actually lower with methadone than with buprenorphine in children 0–17 years old antenatally exposed to either methadone or buprenorphine. Overall, in most cases (15/17 for methadone; 8/15 for buprenorphine) a range of other medications was identified in combination with either methadone or buprenorphine/buprenorphine-naloxone.

Conversely, the ROR values resulting from a comparison of methadone- or buprenorphine-related data *versus all other drugs* were calculated as follows: 

In the case of methadone:

A = methadone with eye disorders = 17;

B = methadone without eye disorders = 2297 − 17 = 2280;

C = all other *psychotropic* drugs with eye disorders, excluding methadone and buprenorphine = 42;

D = all other drugs without eye disorders = 1591 − 42 = 1549

(17/2280)/(42/1549) = 0.00746/0.02711 = 0.27.

In the case of buprenorphine:

A = buprenorphine with eye disorders = 15;

B = buprenorphine without eye disorders = 1199 − 15 = 1184;

C = all other *psychotropic* drugs with eye disorders, excluding methadone and buprenorphine = 42;

D = all other drugs without eye disorders = 1591 − 42 = 1549

(15/1184)/(42/1549) = 0.01267/0.02711 = 0.47.

Specifically, B and D were the total number of all ADRs reported in the 0–17 years age group for each drug, respectively, without the ADRs selected. In the group “all other drugs,” all substances different from opioids (i.e., methadone and buprenorphine) were included. This group comprises the following:

Benzodiazepines: diazepam, alprazolam, clonazepam, lorazepam, bromazepam;

Anticholinergic drugs: biperiden, benztropine, trihexyphenidyl;

Gabapentinoids: pregabalin, gabapentin;

Antipsychotics: quetiapine, olanzapine, clozapine.

In our disproportionality analysis, the RORs for eye disorders resulted in 0.27 (95% CI 0.16–0.48) for methadone and 0.47 (95% CI 0.26–0.85) for buprenorphine, indicating lower reporting odds of eye disorders for these drugs versus the pooled non-opioid comparator group.

### 3.2. Top 20 Medications Associated with Eye Disorders and Congenital Eye Disorders Involving Subjects Aged 0–17 Years Old Exposed In Utero According to the FAERS

**Figure 3:**
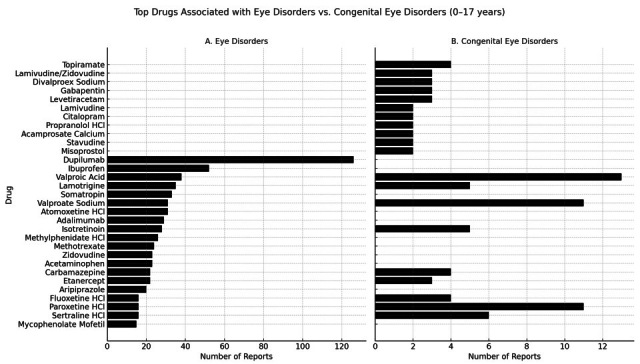
Top 20 medications prescribed during pregnancy and associated with eye disorders/congenital eye disorders in 0–17-year-old individuals according to the FAERS.

Using a complementary methodology, to assess the broader context of paediatric eye disorders potentially related to maternal opioid exposure, the entire FAERS dataset, e.g., up to 2025, was queried for ADRs coded as ‘eye disorders’ and ‘congenital eye disorders’ in children aged 0–17 years. The analysis focused on identifying the most frequently reported drugs. The query yielded a total of 1240 reports, comprising 1143 reports of general eye disorders and 99 reports explicitly involving congenital ocular abnormalities. After excluding duplicate entries and cases lacking evidence or mention of in utero exposure, we identified the drugs most frequently prescribed during pregnancy and associated with eye disorder reports. The leading agents were dupilumab (126 reports), valproic acid/valproate sodium (69 reports), and ibuprofen (52 reports). Other medications recurrently linked to ocular ADRs included antiepileptics (e.g., lamotrigine, carbamazepine), psychotropic agents (e.g., SSRIs, aripiprazole), and immunomodulators (e.g., adalimumab, etanercept). Notably, no opiates/opioids were represented among the top 20 drugs associated with eye disorder reports in this population. Focusing on congenital eye disorders, valproic acid/valproate sodium and paroxetine accounted for the highest number of mentions (24 and 11 reports, respectively). Additional agents included isotretinoin, lamotrigine, and topiramate, further underscoring the established contribution of certain antiepileptic and psychotropic medications to teratogenic ocular risk (Figure 3). Once again, opiates/opioids were absent from the top 20 medications associated with congenital eye defects, suggesting that, within current FAERS data, prenatal opioid exposure does not prominently feature among pharmacological contributors to visual or ocular malformations in offspring.

### 3.3. Analysis of FAERS Data Regarding Eye Disorders in 0–17-Year-Old Children Exposed During Pregnancy to Psychotropics Different from Opiates/Opioids

To better understand current data relating to methadone and buprenorphine, a further descriptive analysis of eye disorders occurring in children prenatally exposed to specific classes of psychoactive medications (e.g., benzodiazepines, anticholinergic drugs, gabapentinoids, and antipsychotics) frequently prescribed in addiction and mental health conditions was carried out here. Although total eye disorder reports were high for drugs like diazepam (2791), alprazolam (2999), and clonazepam (2862), paediatric cases were fewer (ranging 97–242), with only a total of 25 final validated cases identified after de-duplication (Table 2). With regard to anticholinergics, only one confirmed case was identified. Pregabalin (12,170 total) showed no confirmed paediatric cases, whilst gabapentin (3501 total) was associated with five cases after having filtered out duplicates and inconsistencies. Finally, olanzapine, quetiapine, and clozapine were associated with a total of 11 confirmed cases (Table 2; see complete line listings in Supplementary Materials).

## 4. Discussion

### 4.1. Dataset Characteristics and General Findings

To the best of our knowledge, this is the first study using pharmacovigilance data specifically investigating ocular abnormalities in children following prenatal exposure to either methadone or buprenorphine; eye-related data were also compared with those associated with other medications. By systematically analysing spontaneous reports across multiple countries, a range of recurrent ophthalmological events, including nystagmus, strabismus, and reduced visual acuity, aligning with previous clinical observations [14,15,16,17,18] were identified.

### 4.2. Pharmacovigilance Issues

Typical pharmacovigilance studies, with a focus on a range of drug-associated different disturbances, consider for their analysis only those prescribing molecules which present with at least 10 entries [25]. After proper data cleaning, overall numbers of related reports here were indeed relatively small (i.e., 17 for methadone; 15 for buprenorphine), somehow emphasising the rare occurrence of these disturbances in the offspring of mothers being prescribed an opioid substitution treatment. Consistent with current findings, eye disorders such as nystagmus, strabismus, and refractive errors have also been associated with broader neurodevelopmental abnormalities in the context of congenital anomalies [16,26]. Indeed, in utero opioid exposure, particularly to methadone, has been linked to adverse neurodevelopmental outcomes at mid-elementary school age, which may be misinterpreted or misdiagnosed as relating to autism spectrum disorder (ASD) or attention deficit hyperactivity disorder (ADHD) [26]. A further consideration is that most methadone/buprenorphine-reported cases here involved polysubstance use and/or polydrug interventions. Indeed, in the majority of cases, concomitant use of other medications was observed alongside opioid agonist therapy. Specifically, additional pharmacological agents were identified in 15 out of 17 cases involving methadone and in 8 out of 15 cases involving buprenorphine or buprenorphine–naloxone. This indicates that most individuals receiving these treatments were concurrently prescribed or using other medications, suggesting a high prevalence of polypharmacy among patients undergoing maintenance therapy with either methadone or buprenorphine-based regimens. This complexity introduces substantial confounding, as overlapping pharmacological effects, drug–drug interactions, and comorbid maternal conditions complicate both maternal and foetal risk assessment and hinder precise causality attribution in pharmacovigilance data [27,28,29,30]. Overall, in comparing the odds of eye disorders given methadone exposure to the odds of eye disorders given buprenorphine exposure, which represent a relative ROR between two drugs, the ROR resulted in being 0.59, suggesting that the odds of eye disorders were actually lower here with methadone than with buprenorphine in children 0–17 years old antenatally exposed to either methadone or buprenorphine. Conversely, the ROR values resulting from a comparison of methadone- or buprenorphine-related data versus all other psychotropic drugs resulted in being 0.27 and 0.47, respectively. Indeed, a ROR < 1 is a signal of lower reporting odds, not proof of a protective effect. Spontaneous reporting databases measure reporting behaviour, not incidence. Several biases can produce ROR < 1 (or >1): underreporting, differential reporting by drug, confounding by indication, differences in co-medication profiles, age or severity distributions, channelling bias, duplicate reports, or small cell counts. Also, absolute numbers are small for the event of interest (e.g., 17 and 15 events), so results should be interpreted cautiously even when CIs exclude 1. In summary, these results do not imply a causal protective effect. Thus, these findings should be interpreted as the absence of a disproportionality signal for eye disorders with methadone and buprenorphine in our dataset and recommend further investigation (e.g., stratified analyses, review of individual case reports, and pharmacoepidemiological studies) to confirm whether a true difference in risk exists. Previous studies have suggested that prenatal exposure to methadone may confer a greater risk of adverse neurodevelopmental and ophthalmological outcomes compared to buprenorphine, e.g., poorer visual acuity, higher prevalence of strabismus, increased occurrence of nystagmus, and more pronounced refractive errors such as hypermetropia and astigmatism [11,18,31,32]. Current data, however, did not provide any evidence supporting worse outcomes with methadone in relation to buprenorphine; on the contrary, buprenorphine appeared more frequently associated with eye disorders in comparison with methadone. This discrepancy may reflect differences in study design, sample size, follow-up duration, or unmeasured confounding factors such as polysubstance exposure and maternal health. Indeed, methadone and buprenorphine-related eye disorder occurrences have been previously studied whilst comparing children from opiate- or polydrug-dependent mothers versus non-drug using, general population mothers [12]. Whilst rates of misuse may significantly decrease during pregnancy compared to pre-pregnancy intervals [33], some subjects may continue to use multiple drugs and/or alcohol during their gestation. During the first trimester, in fact, the prevalence of past-month drug use among pregnant women may still be up to 22.5% [34]. Similarly, medications such as psychotropics, including benzodiazepines, e.g., diazepam, antipsychotics, e.g., aripiprazole, antidepressants, e.g., paroxetine, and other opioids, e.g., hydrocodone and oxycodone, but also several anti-inflammatory products, antibiotics, and antiepileptic drugs (both topiramate and valproic acid) have been reported. Indeed, several medications taken during pregnancy—including some antiepileptics (valproate, topiramate), anticoagulants (warfarin), and a smaller/less consistent signal for some antidepressants and other drug classes—have been associated with increased risk of congenital eye abnormalities (examples: microphthalmia, coloboma, congenital cataract, optic nerve hypoplasia/atrophy). While isotretinoin and warfarin have well-documented teratogenic ocular effects and certain antiepileptics (valproate, topiramate, carbamazepine) are linked to eye and other craniofacial/neurologic malformations, the strength of evidence for SSRIs is less consistent and mostly points to modest or uncertain increases in some malformations rather than a strong, specific ocular teratogen signal [35]. According to Whelan and Remski [36], in comparison with methadone, buprenorphine is probably best restricted to those with mild–moderate dependence. Hence, one would wonder if mothers having been prescribed with methadone during their pregnancy were indeed presenting with a problematic/severe form of opiated dependency, which is typically associated with high levels of polydrug and alcohol intake during pregnancy. Indeed, the use of buprenorphine in pregnancy has been associated with a lower risk of adverse neonatal outcomes than methadone [37]. Hence, one would hypothesise that the previously reported worse levels of eye-related outcomes in those prescribed with methadone versus buprenorphine [11] were indeed possibly related to the overall more complex clinical scenario observed in methadone-prescribed pregnant patients. Overall, in this complex, polydrug, chronic use scenario it may be very difficult to extrapolate the role of either methadone or buprenorphine in directly causing the eventual occurrence of eye disorders in children.

### 4.3. Comparative Drug Reporting and Absence of Opioid Signals

Considering the top 20 drugs reported in association with eye disorders, dupilumab, valproic acid/valproate sodium, and ibuprofen were those most frequently implicated, alongside other antiepileptics (lamotrigine, carbamazepine), psychotropics (SSRIs, aripiprazole), and immunomodulators (adalimumab, etanercept). Interestingly, opioids—including methadone and buprenorphine—did not appear among the top 20 drugs associated with paediatric eye disorders. The profile of congenital eye disorders differed, with valproic acid/valproate sodium and paroxetine leading the reports, reflecting their well-documented teratogenic potential [32,38]. Additional contributors, such as isotretinoin, lamotrigine, and topiramate, further reinforce the established role of certain antiepileptics and psychotropics in congenital ocular risk. Notably, opioids were again absent from the top 20 drugs associated with congenital eye defects, suggesting that, at least within FAERS voluntary reports, the direct signal for opioid-related congenital ocular abnormalities is minimal.

### 4.4. Potential Mechanisms of Opioid-Related Visual Effects

**Figure 4:**
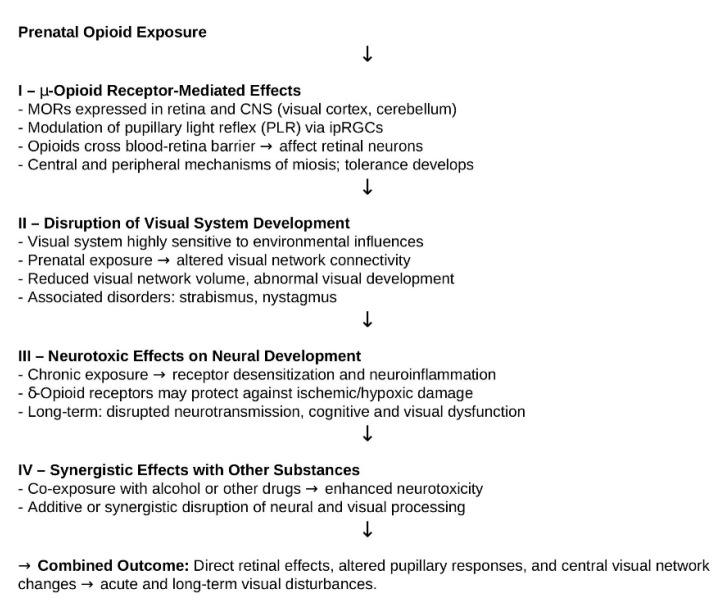
Mechanisms of opioid-related visual effects.

In trying to understand the underlying pharmacological mechanisms contributing to eye disorders in children exposed to opioids during pregnancy, several hypotheses based on receptor interactions and neurodevelopmental effects have been proposed, including the following (Figure 4): (I) μ-opioid receptor-mediated effects due to their abundant expression in the central nervous system, including areas involved in visual processing such as the cerebellum and visual cortex [39]; (II) disruption of the visual system highly sensitive to environmental influences, e.g., prenatal exposure to opioids [12]; (III) neurotoxic effects on neural development [40]; and (IV) synergistic effects with other substances [27,29,39,40]. Regarding the first hypothesis, it has been well-documented that opioid receptors, especially μ-opioid receptors (MORs), are present in the retina and play a role in regulating the pupillary light reflex (PLR).

Both exogeneous and exogeneous opioids can modulate PLR by acting on MORs in intrinsically photosensitive retinal ganglion cells (ipRGCs), which are crucial for non-image-forming visual processes. Opioid agonists can slow or suppress PLR, particularly under dim light, and this effect is consistent with clinical observations of slow pupil constriction in patients on chronic opioid therapy. Opioids can cross the blood–retina barrier and accumulate in the vitreous, directly affecting retinal neurons and ipRGCs [41]. Additionally, opioids are known to cause miosis (pupil constriction) through central and peripheral mechanisms, with tolerance developing over time [42,43,44]. Secondly, opioid receptors are widely distributed in the brain, including regions involved in visual processing and cognitive control. Prenatal opioid exposure has been linked to altered functional connectivity in the primary visual network of infants, with reduced network volume and abnormal visual development, potentially explaining higher rates of visual problems such as strabismus and nystagmus in exposed children [45]. Opioids also modulate neurotransmitter release and neuronal excitability, which can indirectly affect visual perception and processing [46,47,48]. In the third hypothesis, it has been well-documented that opioid receptor activation, particularly δ-opioid receptors, may offer neuroprotection in the retina during ischemic or hypoxic stress by blocking proinflammatory cytokines and glutamate excitotoxicity, stabilising ionic homeostasis, and enhancing antioxidant capacity [49]. However, chronic opioid use can disrupt normal visual and cognitive functions through receptor desensitisation, neuroinflammation, and altered neurotransmitter signalling [50]. Finally, other substances in case of polydrug intake can exploit synergistic effects [27,29,39,40]. Therefore, opioid-related visual effects arise from a combination of direct retinal actions, modulation of pupillary responses, and changes in central visual networks. These mechanisms can lead to both acute and long-term visual disturbances, especially with chronic or prenatal exposure.

### 4.5. Study Limitations

The current pharmacovigilance analysis presented with some limitations, including the following: (i) small sample size, directly related to the rare occurrence of the adverse drug reactions considered here; (ii) lack of denominator, e.g., number of children exposed in utero to methadone/buprenorphine, making it impossible to calculate incidence or rates; (iii) the use of a broad outcome category, with “eye disorders” including very common conditions (e.g., neonatal conjunctivitis) as well as rare ones (e.g., persistent nystagmus, congenital anomalies), which have very different baseline prevalence levels; (iv) underreporting and bias: issues such as underreporting, reporting bias, and incorrect or incomplete information; (v) confounding factors: prematurity, low birth weight, neonatal abstinence syndrome, exposure to other substances, and maternal conditions which could explain ocular effects; and (vi) missing timing of exposure, which is crucial to know when the exposure occurred (e.g., first versus third trimester). The available reports are scarce and often incomplete, reducing the strength of the evidence. Indeed, data retrieved cannot be used to infer causality or risk estimates due to intrinsic limitations including underreporting, reporting bias, lack of denominator data, and frequent polydrug exposure [51,52,53]. Therefore, whilst our findings are hypothesis-generating and may help inform clinicians and researchers, they should primarily be interpreted as evidence of a safety signal that warrants further evaluation. Well-designed prospective studies and registry-based investigations are needed to determine whether opioid exposure in pregnancy truly increases the risk of long-term visual impairment in children.

### 4.6. Research Implications and Clinical Recommendations

Understanding the role of maternal medication exposure to children is essential to improve early detection, preventive strategies, and risk–benefit evaluations of pharmacotherapies used during pregnancy [2]. Also, children who have been prenatally exposed to opioids should be kept under structured and appropriate follow-up beyond the neonatal period and at least until the time of school entry. This is important because developmental, behavioural, and visual difficulties may not be evident in infancy but often emerge or become clinically significant during mid-childhood, when academic, social, and cognitive demands increase [10]. Moreover, monitoring visual and neurological functions in children exposed to opioids should be suggested. Galli et al. [54] monitored annually from the first year of life through 11 years of age two children exposed to methadone during pregnancy, both exhibiting a range of visual difficulties, including ophthalmological problems, oculomotor abnormalities (e.g., nystagmus), and perceptual impairments (reduced visual acuity and diminished contrast sensitivity). While nystagmus and other oculomotor disturbances remained relatively stable over time, gradual improvements were observed in visual acuity and contrast sensitivity.

According to our knowledge, no specific recommendations, restrictions, or precautions have been published by the main teratology information services regarding methadone or buprenorphine possible adverse ocular effects in newborns. To date, in terms of increased risks of congenital malformations, published data on pregnant women exposed to methadone or buprenorphine during the first trimester of pregnancy are reassuring; no specific pattern of birth defects was noted. Also, in relation to functional impairments and neurobehavioral disturbances, which cannot be entirely ruled out, other factors besides these medications could be involved [55,56].

In case of delayed visual maturation, abnormal visual behaviours (poor fixation/tracking), nystagmus, strabismus, excessive tearing, and reduced visual function in prenatally opioid-exposed infants, infants should be referred for paediatric ophthalmology and followed closely during the first year [13,16,38,57]. Also, neurodevelopmental red flags that commonly co-occur (e.g., poor tone, seizures, feeding difficulties) should be monitored as they increase the urgency for neuro-ophthalmic assessment [57]. Practically, at delivery a routine newborn eye screen should be performed, studying red reflex, pupillary response, gross extraocular movements, and ability to fix/track. The infant at risk for neonatal abstinence syndrome or neonatal opioid withdrawal syndrome should be recognised and observed in a setting capable of monitoring and scoring withdrawal [58,59,60]. If signs are normal at birth, clinicians should continue routine well-newborn surveillance and increase vigilance for emerging visual behaviour problems during the first weeks (fixation/tracking) [57]. If any abnormal sign appears, or if the infant requires a pharmacological treatment for neonatal opioid withdrawal syndrome, visual electrophysiology should be considered [16]. During the period between 6 and 12 weeks, an ophthalmology referral is suggested for any abnormal visual behaviour (poor fixation, nystagmus, misalignment) [16]. During the age of 3–6 months, paediatric ophthalmology is required if earlier abnormalities for visual attention and ocular alignment appear [60]. Around 2–3 years, formal visual acuity testing/cycloplegic refraction by an ophthalmologist or orthoptist is needed if there were prior concerns, but they should be considered earlier if strabismus or other abnormal signs appear [60]. Considering the antenatal period, some/preventive recommendations are suggested: firstly, continue evidence-based treatment for opioid use disorder [3], e.g., opioid agonist therapy such as methadone or buprenorphine rather than abrupt cessation or illicit use as treatment reduces maternal and foetal harms; plan delivery at or transfer to a facility that can manage newborns at risk for neonatal abstinence syndrome or neonatal opioid withdrawal syndrome [58]; co-exposures should be reduced where possible (tobacco, alcohol, benzodiazepines) as polydrug exposure worsens neonatal outcomes [58]; and prenatal care through nutrition, folic acid, infection screening, and ultrasounds as indicated should be optimised [3].

Finally, the European Medicines Agency (EMA) emphasises the importance of collecting post-authorization data on medicinal product exposure during pregnancy to assess potential risks to both mother and foetus [61]. The guideline recommends implementing systematic pharmacovigilance, establishing pregnancy exposure registries, and conducting long-term follow-up studies to detect adverse outcomes that may not be identified during pre-marketing clinical trials, as pregnant populations are typically excluded from such studies.

## 5. Conclusions

The current study represents the first pharmacovigilance analysis specifically examining ocular outcomes in children prenatally exposed to either methadone or buprenorphine. The findings suggest the overall lack of potential disproportionality, safety, and signals for eye disorders, including nystagmus, strabismus, and refractive errors, associated with prenatal opioid exposure. In a direct comparison, more signals have been identified in buprenorphine compared to methadone, which may be in contrast with some [11] studies but confirming previous findings [13]. The interpretation of these results is complicated by common co-exposures, polydrug interventions, and underlying maternal comorbidities, which introduce substantial confounding in real-world pharmacovigilance data. Overall, these findings highlight the importance of continued systematic post-marketing surveillance, pregnancy exposure registries, and long-term follow-up studies to better characterise ocular and neurodevelopmental risks associated with prenatal opioid exposure. Furthermore, it is crucial to raise awareness among clinicians about the risks of prenatal opioid exposure, as it may be associated with significant short- and long-term effects on newborns, including increased risks of neonatal opioid withdrawal syndrome, impaired neurodevelopment, and adverse physical health outcomes. Indeed, there is the need for systematic evaluations and follow-up studies investigating opioid- and opiate-exposed infants both immediately after birth and throughout early childhood. Therefore, further larger, prospective, longitudinal, and controlled studies are needed to clarify the independent effects of prenatal opioids/opiates exposure, controlling the potential confounding effect of common co-exposures, polydrug interventions, and maternal comorbidities to guide clinical decision-making regarding opioid maintenance therapy during pregnancy.

## Figures and Tables

**Table 1:** Summary of eye disorder cases in children aged 0–17 years exposed in utero to either methadone or buprenorphine (FAERS dataset up to 1 September 2025): most important findings related to the reactions reported on the total of methadone and buprenorphine cases.

	General Reactions	Eye-Related Reactions	Outcome	Sex	Age
**Methadone: 17 cases**	Foetal Exposure During Pregnancy 6 Maternal Exposure During Pregnancy 5 Small For Dates Baby 3 Developmental Delay 6 Drug Withdrawal Syndrome Neonatal 9	Strabismus 7 Amblyopia 3 Miosis 3 Visus acuity reduced 3 Nystagmus 6 Optic Nerve Hypoplasia 3 Visual Impairment 4 Refraction disorder 3	Other 11 Congenital anomaly 9 Hospitalised 1 Disabled 3 Died 2 Life-threatening 2	F 6 M 4 Not Specified sex 7	1–12 months 9 13–24 months 2–5 years 2 NA 4
**Buprenorphine: 15 cases**	Toxicity To Various Agents 4 Lethargy 3 Somnolence 3 Drug Withdrawal Syndrome 3 Premature Baby 3 Drug Withdrawal Syndrome Neonatal 6 Tremor 4 Agitation 3	Miosis 6	Other 8 Congenital anomaly 5 Hospitalised 14 Disabled 1 Died Life-threatening 4 NA 1	M 7 F 2 Not Specified sex 1	0–12 months 11 13–24 months 1 2–5 years 1

NA = data not available; M = male; F = female.

**Table 2:** Number of total eye disorder-related cases in 0–17-year-old subjects exposed in utero to psychotropic medications; data from the FDA Adverse Event Reporting System.

Substance	Total Eye Disorders	Total Eye Disorders 0–17 Years	Final Validated Cases
**Benzodiazepines**			
Diazepam	2791	240	7
Alprazolam	2999	97	5
Clonazepam	2862	242	7
Lorazepam	2246	117	5
Bromazepam	580	27	1
**Anticholinergic drugs**			
Biperiden	35	6	0
Benztropine	91	15	0
Trihexyphenidyl	166	31	1
**Gabapentinoids**			
Pregabalin	12,170	200	0
Gabapentin	3501	89	5
**Antipsychotics**			
Quetiapine	2054	156	3
Olanzapine	3973	326	7
Clozapine	1354	45	1

## Data Availability

Data derived from public domain resources The data presented in this study are available in the FAERS dataset at https://www.fda.gov/drugs/fdas-adverse-event-reporting-system-faers/fda-adverse-event-reporting-system-faers-public-dashboard (accessed on 11 September 2025).

## Supplementary Materials

The following supporting information can be downloaded at: https://www.mdpi.com/article/10.3390/brainsci15111177/s1, Table S1: Eye disorders cases involving subjects aged 0–17 years old exposed to methadone in utero (FAERS dataset up to 1 September 2025). Table S2: Eye disorders cases involving subjects aged 0–17 years old exposed to buprenorphine, alone or in combination with naloxone, in utero (FAERS dataset up to 1 September 2025). Table S3: Eye disorders cases in patients aged 0–17 years involving psychotropics (FAERS dataset up to 1 September 2025).
