# Supplementary material for: Possible Eye Disorders in Children Prenatally Exposed to Either Methadone or Buprenorphine in Comparison with Other Medications: An Examination of the Food and Drug Administration (FDA) Pharmacovigilance Database
Source: Brain Sci. 2025 Oct 30;15(11):1177. doi: 10.3390/brainsci15111177 (PMC12650041; doi:10.3390/brainsci15111177)
Supplement: Supplementary file 1 [file brainsci-15-01177-s001.zip › brainsci-3938829-supplementary.pdf]

Supplementary materials

Table S1. Eye disorders cases involving subjects aged 0-17 years old exposed to methadone in utero (FAERS dataset up to 1st September 2025)

	Concomitant medications	Reason(s) for Use	Eye-related reactions	Reactions	Outcomes	Sex	Event Date	Patient Age	Reporter Type	Country where Event occurred	Literature Reference
1	Hydroxyzine; Paroxetine	Product Use for unknown indications	Retinopathy	Condition Aggravated;Low Birth Weight Baby;Foetal Exposure During Pregnancy;Maternal Exposure During Pregnancy;Product Substitution	Other Outcomes; Congenital Anomaly	F	2018	1 month	Healthcare Professional	FR	-
2	Pentazocine Lactate;Acetaminophen\Oxycodone Hydrochloride;Oxycodone Hydrochloride;Acetaminophen\Hydrocodone Bitartrate;Diamorphine	Back Pain;Drug Dependence; Affective	Strabismus; Amblyopia	Hypotonia; Speech Disorder Developmental; Tonsillar Hypertrophy;Mental Impairment;Constipation;Behaviour Disorder;Tonsillitis Streptococcal;Tympanic Membrane Perforation;Symbolic Dysfunction;Motor Developmental Delay;Arnold-Chiari Malformation;Premature Baby;Microcephaly;Small For Dates Baby;Sleep Disorder;Autism Spectrum Disorder;Pica;Respiratory Distress;Foetal Exposure During Pregnancy;Developmental Delay;Congenital Scoliosis;Hypoglycaemia;Drug Withdrawal Syndrome Neonatal;Lactose Intolerance	Hospitalized;Disabled;Congenital Anomaly;Other Outcomes	M	2010	NA	Consumer	US	-
3	Acetaminophen\Oxycodone Hydrochloride;Acetaminophen\Hydrocodone; Hydrocodone; Lyrica;Albuterol;Xanax; Combivent;Ambien;Quetiapine Fumarate;Oxygen;Promethazine Hydrochloride	Affective Disorder;Arthralgia;Asthma;Back Pain;Chronic Obstructive Pulmonary Disease;Drug Dependence;Hypersensitivity;Seizure;Spinal Pain	Amblyopia	Drug Withdrawal Syndrome Neonatal;Low Birth Weight Baby;Upper Respiratory Tract Infection;Ear Infection;Developmental Delay;Tachypnoea;Foetal Exposure During Pregnancy;Learning Disorder;Hyperbilirubinaemia;Premature Baby	Hospitalized;Other Outcomes	M	2017	NA	Healthcare Professional	US	
4	Acetaminophen\Hydrocodone	Product Use for unknown indication	Miosis	Respiratory Rate Decreased;Unresponsive To Stimuli;Somnolence;Cyanosis;Exposure Via Breast Milk;Overdose; Bradypnoea; Overdose	Other Outcomes	NA	NA	13 months	Healthcare Professional	US	West PL, Mckeown NJ, Hendrickson RG.. Methadone overdose in a breast-feeding toddler. Clin Toxicol (Phila). 2009;47:721

5		Product Use for unknown indication	Miosis	Hypertransaminasaemia;Bradycardia;Hydrocephalus;Intracranial Pressure Increased;Hypotension;Hemiparesis;Cerebellar Infarction;Superior Sagittal Sinus Thrombosis;Blood Urea Increased;Coma;Nasal Congestion;Apnoea;Dysphagia;Encephalopathy;Anisocoria;Dilated Cardiomyopathy;Leukocytosis;Blood Creatinine Increased	Other Outcomes;Hospitalized;Life Threatening	M	2019	21 months	Healthcare Professional	US	Ly S, Connolly K, Vargas V. Rapid decompensation of a toddler that tested positive for methadone. J-Invest-Med 2019;67(1):278 abstr. 493.
6	Clonazepam; Aripiprazole	Anxiety;Arthritis;Bipolar Disorder;Intervertebral Disc Protrusion	Strabismus;Visual Acuity Reduced	Craniosynostosis;Jaundice;Transient Tachypnoea Of The Newborn;Auditory Disorder;Drug Withdrawal Syndrome Neonatal;Premature Baby;Hypoglycaemia;Hyperbilirubinaemia;Foetal Exposure During Pregnancy; Cardiac Murmur;Pulmonary Artery Stenosis Congenital;Jaundice Neonatal;Auditory Disorder;Preterm Premature Rupture Of Membranes;;Atrial Septal Defect;Transient Tachypnoea Of The Newborn;Group B Streptococcus Neonatal Sepsis	Congenital Anomaly;Other Outcomes; Hospitalized	M	2017	0	Consumer	US	-
7	Morphine	Feeling Abnormal;Off Label Use;Product Used For Unknown Indication	Pupil Fixed	Crying;Pulmonary Oedema;Heart Rate Increased;Cyanosis;Brain Oedema;Toxicity To Various Agents;Pulmonary Congestion;Brain Death; Foetal Exposure During Pregnancy;Respiratory Arrest	Died	F	2014	3 months	Healthcare Professional	CA	Lewington LE, Shaffer C, Ornstein AE.. Paediatric methadone ingestions: An under-recognized form of child maltreatment?. Paediatr Child Health. 2014;19 (3):139-140
8	Diazepam; Diamorphine	Product Used For Unknown Indication	Visual Acuity Reduced; Nystagmus;Strabismus; Optic Nerve Hypoplasia;Visual Impairment	Developmental Delay;Maternal Drugs Affecting Foetus; Congenital Central Nervous System Anomaly	Other Outcomes;Congenital Anomaly	NA	2012	6 months	Healthcare Professional	GB	Gupta M,Mulvihill A,Lascaratos G,Fleck B,George N. Nystagmus and reduced visual acuity secondary to drug exposure in utero: Long-term follow-up. Journal of Pediatric Ophthalmology and Strabismus 2012 Feb;49:1:58-63.

9	Buprenorphine Hydrochloride\Naloxone Hydrochloride	Drug Dependence	Amblyopia	Rash;Dermatitis Diaper;Developmental Hip Dysplasia; Muscle Rigidity;Foetal Exposure During Pregnancy;Liver Disorder;Drug Withdrawal Syndrome Neonatal	Other Outcomes;Congenital Anomaly	F	2010	0	Consumer	US	-
10	Heroin; Diamorphine;Methadone Hydrochloride;Cannabis Sativa Subsp. Indica Top;Unspecified Ingredient;Alcohol	Maternal Exposure During Pregnancy	Nystagmus;Myopia;Strabismus Astigmatism; Maculopathy;Myopia;Vision Abnormal Neonatal;Visual Evoked Potentials Abnormal	Developmental Delay;Dysmorphism; Premature Baby;Drug Withdrawal Syndrome Neonatal; Congenital Visual Acuity Reduced;Hypermetropia;	Other Outcomes	NA	2010	9 months	Healthcare Professional	GB	Hamilton R,McGlone L,MacKinnon J,Russell H,Bradnam M,Mactier H. Ophthalmic, clinical and visual electrophysiological findings in children born to mothers prescribed substitute methadone in pregnancy. British Journal of Ophthalmology 2010 Jun;94:6:696-700.
11	Amphetamine Aspartate\Amphetamine Sulfate\Dextroamphetamine Saccharate\Dextroamphetamine Sulfate;Diazepam;Methadone Hydrochloride;Dihydrocodeine;Diazepam; Nitrazepam; Heroin	Product Use for Unknown Indication; Maternal Exposure During Pregnancy	Visual Impairment; Nystagmus; Optic Nerve Hypoplasia; Refraction Disorder; Visual Impairment;Visual Acuity Reduced	Potentiating Drug Interaction;Developmental Delay;Maternal Exposure During Pregnancy;Potentiating Drug Interaction;Drug Withdrawal Syndrome neonatalAttention Deficit Hyperactivity Disorder;Maternal Drugs affecting foetus; Foetal Alcohol Syndrome; Microcephaly	Congenital Anomaly;Other Outcomes	NA	2010	6 months	Healthcare Professional	GB	Mulvihill AO, Cackett PD, George ND, Fleck BW. Nystagmus secondary to drug exposure in utero. Br J Ophthalmol. 2007; 91; 613-615
12	Diazepam; Unspecified Ingredient	Product Used For Unknown Indication	Congenital Nystagmus;Visual Impairment;Strabismus; Optic Nerve Hypoplasia	Attention Deficit Hyperactivity Disorder;Posture Abnormal; Developmental Delay;Maternal Drugs Affecting Foetus;Posture Abnormal;Developmental Delay;Microcephaly;Drug Withdrawal Syndrome Neonatal	Congenital Anomaly;Other Outcomes	NA	2008	4 years	Healthcare Professional	NA	-
13	Erythromycin;Unspecified Ingredient;Ceftriaxone; Acyclovir;Dexamethasone;Amoxicillin	Encephalitis	Visual Impairment	Encephalitis; Heart Rate Increased;Cerebral Palsy;Toxicity To Various Agents;White Blood Cell Count Increased;Areflexia;Dystonia;Hypotension;Pupil Fixed;Dyspnoea;Coma Scale Abnormal;Hypothermia;Metabolic Acidosis	Disabled;Life threatening;Hospitalized	F	2008	3 years	Healthcare Professional	GB	-

14	Diazepam;Amitriptyline Hydrochloride	Product Used For Unknown Indication	Refraction Disorder;Strabismus;Nystagmus;	Maternal Exposure During Pregnancy; Drug Interaction;Developmental Delay;Neonatal Disorder;Drug Withdrawal Syndrome Neonatal	Other outcomes	NA	2008	12 months	Healthcare Professional	NA	-
15	Dihydrocodeine;Diazepam; Amphetamine	Maternal Exposure During Pregnancy	Visual Impairment;Nystagmus ;Congenital Nystagmus;Hypermetropia;Vision Abnormal Neonatal	Maternal Exposure During Pregnancy;Developmental Delay	Congenital Anomaly	NA	2007	6 months	Healthcare Professional	NA	-
16	Heroin;Haloperidol;Lorazepam; Flunitrazepam;Amitriptyline Hydrochloride;Haloperidol;Diamorphine;Codeine;Biperiden Hydrochloride	Maternal Exposure During Pregnancy	Strabismus	Developmental Coordination Disorder;Small For Dates Baby; Psychomotor Skills Impaired	Other Outcomes;Congenital Anomaly	F	1994	0	Healthcare Professional	DE	-
17			Miosis	Circulatory Collapse;Seizure;Drug Withdrawal Syndrome Neonatal;Maternal Exposure During Pregnancy;Intracranial Pressure Increased;Hypotension;Overdose;Cardiac Disorder;Stupor;Respiratory Depression	Other Outcomes;Life Threatening;Hospitalized; Died	F	2005	5 months	Healthcare Professional	NA	-

Abbreviations: CA: Canada; DE: Germany; F: female; FR: France; GB: Great Britain; M: male; NA: not available; US: United States of America

Table S2. Eye disorders cases involving subjects aged 0-17 years old exposed to buprenorphine, alone or in combination with naloxone, in utero (FAERS dataset up to 1st September 2025)

	**Concomitant medications**	**Reason(s) for Use**	**Eye-related reactions**	**Reactions**	**Outcomes**	**Sex**	**Event Date**	**Patient Age**	**Reporter Type**	**Country where Event occurred**	**Literature Reference**
1	Naloxone	Product Used For Unknown Indication	Miosis	Drug Withdrawal Syndrome;Lethargy;Somnolence;Feeding Disorder	NA	M	NA	14 days	Healthcare Professional	US	Anderson P.O. Opioid Use Disorder During Breastfeeding. Breastfeeding Medicine. 2023;18(6):410-412
2	Oxycodone;Tapentadol ;Acetaminophen	Back Pain	Visual impairment	Ear Infection;Brain Injury;Premature Baby;Drug Dependence;Cardiac Murmur;Attention Deficit Hyperactivity Disorder;Behaviour Disorder;Mental Disorder;Emotional Disorder;Pain;Respiratory Tract Infection;Developmental Delay;Speech Disorder;Cognitive Disorder;Gastrooesophageal Reflux Disease;Low Birth Weight Baby;Weight Gain Poor;Eating Disorder;Congenital Musculoskeletal Disorder;Tremor;Foetal Exposure During Pregnancy;Drug Withdrawal Syndrome Neonatal;Musculoskeletal Stiffness;Language Disorder;Disturbance In Attention;Dysphagia; Insomnia	Hospitalized;Congenital Anomaly	NA	2012	NA	Consumer	US	-

3		-	Eye Disorder; Miosis	Toxicity To Various Agents;Mental Disorder;Lethargy;Dyspnoea;Infantile Apnoea;Unresponsive To Stimuli	Other Outcomes; Hospitalized;Life Threatening	M	NA	22 MTH	Healthcare Professional	US	Hedstrom, R.. Analeptic dose of flumazenil in a toddler without benzodiazepine exposure. Clinical Toxicology. 2020;58(11):1144-5
4	Ranitidine;Acetaminophen\Hydrocodone Bitartrate;Alprazolam; Clonazepam;Diazepam;Naloxone; Acetaminophen\Butabarbital\Caffeine;Morphine;Gabapentin;Promethazine;Oxycodone;Betamethasone	Anxiety;Migraine;Pain		Foetal Hypokinesia;Paternal Exposure During Pregnancy;Premature Baby;Neonatal Respiratory Distress Syndrome;Tremor;Dyspnoea;Drug Withdrawal Syndrome Neonatal;Poor Feeding Infant;Gastrooesophageal Reflux Disease;Exposure Via Breast Milk;Infantile Vomiting;Foetal Growth Restriction;Bradycardia Foetal;Hearing Disability;Sepsis;Foetal Exposure During Pregnancy;Hyperbilirubinaemia;Behaviour Disorder;Low Birth Weight Baby;Crying	Other Outcomes; Hospitalized	M	2016	0 YR	Consumer	US	-
5		Drug Use Disorder	Miosis	Toxicity To Various Agents;Hypoglycaemia;Exposure Via Breast Milk;Drug Withdrawal Syndrome Neonatal;Somnolence;Lethargy;Poor Feeding Infant;Bradycardia;Selective Eating Disorder;Coma Scale Abnormal	Hospitalized;Other Outcomes	M	NA	2 WEEK	Healthcare Professional	US	Dorey A, Owen K, Chenoweth J, Catlin J, Ford J. Possible buprenorphine toxicity in a breastfeeding neonate. Clin Toxicol (Phila). 2019;57(10):889-890 (36)
6	Diamorphine;Gabapentin;Tobacco leaf	Product Used For Unknown Indication	Blindness;Eyelid Ptosis; Miosis	Foetal Exposure During Pregnancy;Gastrooesophageal Reflux Disease;Micrognathia;Hydronephrosis;Congenital Multiplex Arthrogryposis;Vomiting;Drug Withdrawal Syndrome Neonatal;Cerebral Ventricle Dilatation;Palatal Disorder;Hypoxic-Ischaemic Encephalopathy;Developmental Hip Dysplasia;Sepsis;Knee Deformity;Neonatal Respiratory Distress Syndrome;High-Pitched Crying;Cerebral Disorder;Deafness Bilateral;Anorectal Disorder;Toxicity To Various Agents;Drug Interaction;Ear Deformity Acquired;Feeding Intolerance	Congenital Anomaly;Life Threatening ;Other Outcomes; Disabled;Hospitalized	M	NA	35 WEEK	Healthcare Professional	US	Maddox TR, Haas J, Andrews L, Miller B, Davies TH.. Abnormal Presentation of Hypoxic Ischemic Encephalopathy Attributed to Polysubstance Exposure. Am J Case Rep. 2019;20:1715-1718
7	Diazepam	Product Used For Unknown Indication	Miosis	Hypopnoea;Somnolence;Urinary Incontinence;Encephalitis;Overdose;Unresponsive To Stimuli;Loss Of Consciousness	Life Threatening ;Other Outcomes; Hospitalized	M	NA	5 YR	Healthcare Professional	US	Caravati E. Martin, Drapkin Zachary, Plumb Jennifer, Szadklowski Matthew A.. Acute Cerebellitis in a 5-Year-Old Child. Pediatric Emergency Care. 2018;34(9):175-177
8		-	Mydriasis	Hyperreflexia;Foetal Exposure During Pregnancy;Yawning;Tremor;Sneezing;Agitation;Hyperhidrosis;Exposure Via Breast Milk;Myoclonus;Insomnia;Withdrawal Syndrome	Hospitalized;Other Outcomes	F	NA	4 months	Healthcare Professional	US	Elladki, et al. H. Buprenorphine withdrawal in an infant after cessation of breastfeeding: A case report and review of the literature.. Pharmacotherapy. 2011 OCT 01;31:.
9		Drug Dependence	Exudative Retinopathy	Cardiac Murmur;Irritability;Premature Baby	Congenital Anomaly;Other Outcomes; Hospitalized	M	2008	0 YR	Healthcare Professional	US	-
10	Carbamazepine;Clorazepate Dipotassium;Unspecified Ingredient	-	Eye Movement Disorder;	Crying;Coordination Abnormal;Urine Cannabinoids Increased;Foetal Heart Rate Deceleration Abnormality;Small For Dates Baby;Drug Withdrawal Syndrome Neonatal;Opiates;Foetal Growth Restriction;Jaundice Neonatal;Tremor Neonatal;Sneezing;Pyrexia;Anticonvulsant Drug Level;Body Temperature Decreased;Body Height Below Normal;Weight Decreased;Maternal Exposure During Pregnancy;Agitation	Hospitalized;Other Outcomes	F	2006	1 DAY	Healthcare Professional	FR	-
11	Fluoxetine Hydrochloride, Bromazepam	Product Used For Unknown Indication	Blepharophimosis;Hypertelorism	Congenital Facial Diplegia;Talipes;Maternal Exposure During Pregnancy;Neonatal Seizure;Infantile Apnoea;Iiird Nerve Disorder;Otospondylomegaepiphyseal Dysplasia;Neonatal Infection;High Arched Palate;Dysmorphism;Cleft Uvula;Hypotonia Neonatal;Neonatal Aspiration	Hospitalized;Congenital Anomaly	F	2005	0 YR	Healthcare Professional	FR	-

12		Drug Detoxification	Strabismus	Tremor Neonatal;Jaundice Neonatal;Congenital Anomaly;Maternal Exposure During Pregnancy;Plagiocephaly;Agitation Neonatal	Hospitalized;Congenital Anomaly	M	2002	1 DAY	Not Specified	NA	-
13		Drug Dependence	Lacrimation Increased	Tremor;Maternal Drugs Affecting Foetus;Drug Withdrawal Syndrome Neonatal	Hospitalized	F	1999	1 DAY	Not Specified	NA	-
14		Drug Dependence	Miosis	Vomiting;Benign Congenital Hypotonia;Syncope;Hypertonia;Neonatal Respiratory Depression;Maternal Drugs Affecting Foetus;Crying;Cyanosis;Neonatal Respiratory Arrest;Infantile Vomiting;Coma;Hypoglycaemia;Neonatal Seizure;Dyskinesia;Pallor;Bradycardia	Hospitalized;Life Threatening	F	1999	1 DAY	Healthcare Professional	NA	-
15	Naloxone, Fluoxetine; Aripiprazole, Alprazolam	Drug Withdrawal Syndrome	Lacrimation Increased	Rhinorrhoea;Pruritus;Hyperventilation;Vomiting;Nausea;Diarrhoea;Hallucination;Confusional State;Presyncope;Heart Rate Increased	Hospitalized	F	2025	6 WEEKS	Consumer	NA	

Abbreviations: F: female; FR: France; M: male; NA: not available; US: United States of America

Table S3: Eye disorders cases in patients aged 0-17 years involving psychotropics (FAERS dataset up to 01st September 2025)

**Eye disorders cases in patients aged 0-17 years (FAERS dataset up to 01st September 2025)**												
	**Concomitant ingredients**	**Reason for Use**	**Eye-related reactions**	**Reactions**	**Serious**	**Outcomes**	**Sex**	**Event Date**	**Patient Age**	**Reporter Type**	**Country where Event occurred**	**Literature Reference**
**Benzodiazepines**												
1 Diazepam	Valproic Acid, Aripiprazole	Foetal Exposure During Pregnancy	Astigmatism	Foetal Anticonvulsant Syndrome; Hypothyroidism;Dysmorphism;Cardiac Murmur;Coloboma;Syndactyly;Hypotonia;Congenital Skin Dimples;Motor Developmental Delay;Foetal Exposure During Pregnancy;Hyperreflexia;Deafness Unilateral	Serious	Congenital Anomaly	Female	2024	17 months	Healthcare Professional	NA	.
2 Diazepam	Gabapentin;Clonazepam;Acetaminophen\Butabarbital\Caffeine;Buprenorphine Hydrochloride\Naloxone Hydrochloride;Oxycodone;Ranitidine Hydrochloride;Acetaminophen\Hydrocodone Bitartrate;Alprazolam;Buprenorphine Hydrochlori	Anxiety; Migrain; Pain	Eye Disorder	Low Birth Weight Baby;Foetal Hypokinesia;Poor Feeding Infant;Tremor;Infantile Vomiting;Paternal Exposure During Pregnancy;Crying;Neonatal Respiratory Distress Syndrome;Dyspnoea;Sepsis;Foetal Growth Restriction;Foetal Exposure During Pregnancy;Behaviour Disorder;Hyperbilirubinaemia;Bradycardia Foetal;Premature Baby;Drug Withdrawal Syndrome Neonatal;Hearing Disability;Gastrooesophageal Reflux Disease;Exposure Via Breast Milk	Serious	Hospitalized; Other outcomes	Male	2016	0	Consumer	US	-

	de;Morphine Sulfate;Betamethasone;Promethazine Hydrochloride											
3 Diazepam	Hydroxyzine Hydrochloride;Valproate Sodium;Albuterol Sulfate;Potassium Chloride	Product Used For Unknown Indication	Visual Impairment Myopia;	Tonsillectomy;Speech Disorder Developmental; Sensory Processing Disorder;Foot Deformity;Foetal Exposure During Pregnancy;Communication Disorder;Dyspnoea;Adenoidectomy;Dysgraphia;Sleep Disorder;Inguinal Hernia;Language Disorder;Stereotypy;Anxiety;Disturbance In Social Behaviour;Oesophagitis;Neurodevelopmental Disorder;Behaviour Disorder;Premature Baby;Feeding Disorder;Echolalia;Aphasia;Respiratory Distress;Learning Disorder;Emotional Disorder Of Childhood;Autism Spectrum Disorder;Dysmorphism;Personal Relationship Issue	Serious	Other outcomes; Congenital Anomaly;Hospitalized	Male	2004	15 days	Healthcare Professional	FR	-
4 Diazepam	Methadone	Product Used For Unknown Indication	Nystagmus; Visual Acuity Reduced; Strabismus	Congenital Central Nervous System Anomaly; Developmental Delay;;Maternal Drugs Affecting Foetus	Serious	Oher Outcomes	NA	2013	6 months	Healthcare Professional	NA	-
5 Diazepam	Methadone, Heroin	Foetal Exposure During Pregnancy	Strabismus Congenital;Congenital Nystagmus;Visual Impairment;	Maternal Drugs Affecting Foetus	Serious	Congenital Anomaly	Male	2012	18 months	Healthcare Professional	GB	Gupta M,Mulvihill A,Lascaratos G,Fleck B,George N. Nystagmus and reduced visual acuity secondary to drug exposure in utero: Long-term follow-up. Journal of Pediatric Ophthalmology and Strabismus 2012 Feb;49:1:58-63.
6 Diazepam	Methadone; Nitrazepam	Foetal Exposure During Pregnancy	Visual Impairment; Congenital Nystagmus;Optic Nerve Hypoplasia;Refraction Disorder	;Maternal Exposure During Pregnancy; Foetal Alcohol Syndrome;Microcephaly;Potentiating Drug Interaction;Developmental Delay	Serious	Congenital Anomaly; Other outcomes	NA	2010	9 months	Consumer	GB	-
7 Diazepam	Methadone	NA	StrabismusNystagmus;Visual Impairment	Developmental Delay;Posture Abnormal	Serious	Congenital Anomaly; Other outcomes	NA	2008	4 years	NA	GB	Mulvihill, Cackett, P, George N, Fleck B. Nystagmus Secondary to Drug Exposure in Utero. Br. J of Oophtalmology 2007 May;91(5):613-615.
1 Alprazolam	Gabapentin;Clonazepam;Acetaminophen\Butabarbital\Caffeine;Acetaminophen\Butalbital\Caffeine;Buprenorphine Hydrochloride\Naloxone Hydrochloride;Oxycodone;Ranitidine Hydrochloride;Acetami	Anxiety;Migraine;Pain	Eye Disorder	Low Birth Weight Baby;Foetal Hypokinesia;Poor Feeding Infant;Tremor;Infantile Vomiting;Paternal Exposure During Pregnancy;Crying;Neonatal Respiratory Distress Syndrome;Dyspnoea;;Sepsis;Foetal Growth Restriction;Foetal Exposure During Pregnancy;Behaviour Disorder;Hyperbilirubinaemia;Bradycardia Foetal;Premature Baby;Drug Withdrawal Syndrome Neonatal;Hearing Disability;Gastrooesophageal Reflux Disease;Exposure Via Breast Milk	Serious	Hospitalized; Other outcomes	Male	2016	0	NA	US	-

	nophen\Hydrocodone Bitartrate;Alprazolam;Buprenorphine Hydrochloride;Ranitidine;Morphine Sulfate;Betamethasone;Buprenorphine;Promethazine Hydrochloride;Gabapentin											
2 Alprazolam	Imipramine; Nitrazepam; Flunitrazepam	Ill-defined disorder	Eye disorder	Joint Hyperextension;Maternal Exposure During Pregnancy;Tremor;Opisthotonus;Selective Eating Disorder;Hypertonia Neonatal;Neonatal Seizure;Drug Withdrawal Syndrome Neonatal;Stridor;Gaze Palsy	Serious	Hospitalized	Male	2005	1 day	Healthcare Professional	NA	-
3 Alprazolam	Imipramine Hydrochloride;Alprazolam;Amoxapine;Paroxetine Hydrochloride;Unspecified Ingredient	Panic Disorder; Prophylaxia	Visual Impairment	Maternal Drugs Affecting Foetus;Use Of Accessory Respiratory Muscles;Body Temperature Increased;Talipes;Hypotonia Neonatal;Pharyngeal Oedema;Apgar Score Low;Tremor;Neonatal Disorder;Skin Disorder;Neonatal Asphyxia; Dysphonia;Anaemia;Nervous System Disorder;Premature Baby;Oedema	Serious	Other outcomes	Female	2002	0	Consumer	JP	-
4 Alprazolam			Eye disorder	Maternal Drugs Affecting Foetus;Deafness;Neonatal Disorder;Pregnancy;Floppy Infant;Drug Withdrawal Syndrome Neonatal;Gaze Palsy;Tremor Neonatal	Serious	Disabled; Other outcomes	Female	2002	0	Healthcare Professional	NA	-
5 Alprazolam	Haloperidol;Clomipramine Hydrochloride		Eye disorder	Bradycardia;Abdominal Pain;Strabismus;Cyst;Gastrointestinal Malformation;Bronchitis;Growth Retardation;Neonatal Disorder;Maternal Drugs Affecting Foetus;Developmental Coordination Disorder;Benign Congenital Hypotonia	Serious	Hospitalized	Male	2001	1 day	Healthcare Professional	NA	-
1 Clonazepam	Gabapentin;Acetaminophen\Butabarbital\Caffeine;Acetaminophen\Butalbital\Caffeine;Buprenorphine Hydrochloride\Naloxone Hydrochloride;Oxycodone;Diazepam;Ranitidine Hydrochloride;Acetaminophen\Hydrocodone Bitartrate;Alprazolam;Ranitidine;Di	Anxiety; Migrain; Pain	Eye disorder	Low Birth Weight Baby;Foetal Hypokinesia;Poor Feeding Infant;Tremor;Infantile Vomiting;Paternal Exposure During Pregnancy;Crying;Neonatal Respiratory Distress Syndrome;Dyspnoea;Eye Disorder;Sepsis;Foetal Growth Restriction;Foetal Exposure During Pregnancy;Behaviour Disorder;Hyperbilirubinaemia;Bradycardia Foetal;Premature Baby;Drug Withdrawal Syndrome Neonatal;Hearing Disability;Gastrooesophageal Reflux Disease;Exposure Via Breast Milk	Serious	Hospitalized; Other outcome	Male	2016	0	Consumer	US	-

	azepam;Clonazepam;Morphine Sulfate;Betamethasone;Promethazine Hydrochloride;Gabapentin; Hydrochloride;Acetaminophen\Hydrocodone											
2 Clonazepam	Methadone; Aripiprazole	Anxiety;Arthritis;Bipolar Disorder;Intervertebral Disc Protrusion	Strabismus	Drug Withdrawal Syndrome Neonatal;Foetal Exposure During Pregnancy;Craniosynostosis;Auditory Disorder;Transient Tachypnoea Of The Newborn; Premature Baby;Jaundice;Hypoglycaemia;Hyperbilirubinaemia;	Serious	Congenital anomaly; Other outcomes	Make	2017	0	Healthcare Professional	US	
3 Clonazepam	Valproate Sodium;Lamotrigine	Epilepsy; Prophylaxys	Strabismus; Congenital Myopia; Ophthalmia Neonatorum; Hypertelorism; Astigmatism; Conjunctivitis; Deformity Of Orbit	Gastrooesophageal Reflux Disease;Social Avoidant Behaviour;Ligament Laxity;Bronchitis;Intellectual Disability;Joint Laxity;Language Disorder; Talipes;Motor Dysfunction;Foetal Exposure During Pregnancy;Atrophy;Neonatal Asphyxia;Drooling;Hypotonia Neonatal;Delayed Fontanelle Closure;Otitis Media Acute;Somnolence;Balance Disorder;Neonatal Respiratory Distress; Aminoaciduria;Developmental Delay;Left-To-Right Cardiac Shunt;Dysmorphism;Bronchiolitis;Meconium In Amniotic Fluid;Ear Disorder;Intelligence Test Abnormal;Nasal Disorder;Psychomotor Retardation;Speech Disorder;Disturbance In Attention; Congenital Cerebral Cyst;Conjunctivitis;Acidosis; Oropharyngeal Discomfort;Ventricular Septal Defect;Respiratory Disorder Neonatal;Growth Retardation;Dysgraphia;Abdominal Distension;Congenital Sleep Disorder;Speech Disorder Developmental; Abdominal Distension; Speech Disorder Developmental; Dysmorphism;Small For Dates Baby	Serious	Other Outcomes; Hospitalized;Congenital Anomaly	Male	2020	1 month	Healthcare Professional	FR	
4 Clonazepam	Acetaminophen\Codeine Phosphate; Hydrocodone;Nitrofurantoin;Docusate Sodium;Amoxicillin;Topiramate;Loperamide;Levetiracetam		Eye Disorder	Metabolic Acidosis;Respiratory Distress;Cleft Lip And Palate;Conductive Deafness;Hypoacusis;Nose Deformity;Patent Ductus Arteriosus;Emotional Disorder;Eustachian Tube Dysfunction;Otitis Media Chronic; Developmental Delay;Hypotonia;Congenital Foot Malformation; Foetal Exposure During Pregnancy	Serious	Other Outcomes; Hospitalized;Congenital Anomaly	Male	2007	0	Healthcare Professional	US	
5 Clonazepam	Valproate Sodium;Carbamazepine	Maternal Exposure During Pregnancy	Myopia	Foetal Anticonvulsant Syndrome;Dysmorphism;Atrial Septal Defect;Neurodevelopmental Disorder;Extremity Contracture; Deafness;Kidney Malformation	Serious	Other outcomes	Female	1996	0	Consumer	GB	
6 Clonazepam	Promethazine Hydrochloride;Trihexyphenidyl Hydrochloride;Fluvoxamine Maleate;Risperidone;Clonazepam;	Schizophrenia	Eye Movement Disorder	Somnolence;Irritability;Drug Withdrawal Syndrome Neonatal;Pyrexia;Liver Function Test Abnormal;Tremor;Hypotonia	Serious	Other Outcomes; Congenital Anomaly	Female	2006	0	Healthcare Professional	JP	

	Quetiapine Fumarate;Flunitrazepam											
7 Clonazepam	Valproate Sodium	Maternal Exposure During Pregnancy	Congenital Eye Disorder	Bicuspid Aortic Valve;Limb Deformity;Nipple Disorder;Blood Phosphorus Increased;Atrial Septal Defect;Congenital Anomaly;Congenital Hand Malformation;Low Set Ears;Congenital Nail Disorder;Congenital Nose Malformation; Maternal Exposure During Pregnancy;Pupillary Disorder;Blood Calcium Decreased;Hypospadias;Foetal Anticonvulsant Syndrome;Congenital Aplasia;Dysmorphism;Anterior Chamber Cleavage Syndrome	Serious	Congenital Anomaly	Male	2005	1 month	Healthcare Professional	NA	
1 Bromazepam	Morphine Sulfate;Nicotine;Diamorphine	Maternal Exposure During Pregnancy	Strabismus	Neutropenia;Hypermetropia;Nausea;Blood Disorder;Social Problem;Macrocytosis;Maternal Exposure During Pregnancy;Urine Abnormality;Neonatal Disorder;Drug Withdrawal Syndrome Neonatal;Hepatosplenomegaly;Abnormal Behaviour;Premature Baby;Torticollis;Cytomegalovirus Infection;Selective Eating Disorder;Strabismus;Developmental Delay;Movement Disorder;Restlessness;Communication Disorder;Abdominal Distension;Foot Deformity	Serious	Hospitalized	Female	1995	1 day	Healthcare Professional	NA	
1 Lorazepam	Paroxetine Hydrochloride; Bupivacaine Hydrochloride	Product Used For Unknown Indication	Eye disorder	Maternal Exposure During Pregnancy;Brain Stem Ischaemia;Neonatal Asphyxia;Hypoxia;Unresponsive To Stimuli;Apnoea;Mydriasis;Muscle Tightness	Serious	Other outcomes	Female	2007	0	Healthcare Professional	JP	
2 Lorazepam	Biperiden Hydrochloride;Haloperidol;Diamorphine;Methadone Hydrochloride;Amitriptyline Hydrochloride;Flunitrazepam;Codeine		Strabismus	Maternal Exposure During Pregnancy; Small For Dates Baby;Developmental Coordination Disorder	Serious	Other Outcomes; Congenital Anomaly	Female	1994	0	Healthcare Professional	DE	
3 Lorazepam	Venlafaxine	Drug withdrawal Syndrome; Depression	Photophobia	Irritability;Vomiting Projectile;Rebound Effect;Muscle Rigidity;Tremor Neonatal;Muscle Spasms;Foetal Distress Syndrome;Therapy Non-Responder;Poor Sucking Reflex;Hyporeflexia;Laryngeal Disorder;Selective Eating Disorder;Caesarean Section;Nasal Flaring;Hyperaesthesia;Maternal Exposure During Pregnancy;Nervous System Disorder;Joint Hyperextension;Therapeutic Product Effect Decreased;Aspiration;Infantile Diarrhoea;Drug Withdrawal Syndrome Neonatal; Restlessness;Caesarean Section;Diaphragmatic Disorder;Vomiting Projectile;Drug Withdrawal Syndrome Neonatal;Rebound Effect;Photophobia;Neonatal Disorder;Foetal Distress Syndrome;Toxicity To Various Agents;Muscle Rigidity;Agitation Neonatal; Hyperaesthesia;Drug Ineffective;Joint Hyperextension;Neonatal Tachypnoea;Nervous System Disorder;Irritability;Blood Bilirubin Increased;Hyperacusis;Nasal Flaring	Serious	Disabled;Other Outcomes; Hospitalized	Male	2006	1 day	Healthcare Professional	NA	-
4 Lorazepam			Pupillary Disorder;Optic Nerve Disorder; Blindness Congenital;Iris Disorder	Maternal Drugs Affecting Foetus	Serious	Congenital Anomaly	Male	2000	0	Healthcare Professional	NA	

5 Lorazepam	Quetiapine Fumarate;Perphenazine;Trazodone Hydrochloride;Levomepromazine; Citalopram Hydrobromide;Zopiclone	Depression	Eye Disorder	Neonatal Disorder;Neonatal Respiratory Depression;Central-Alveolar Hypoventilation;Hypoventilation Neonatal;Congenital Anomaly;Hypotonia Neonatal;Maternal Drugs Affecting Foetus	Serious	Congenital Anomaly;Hospedalized	Female	2002	0	Healthcare Professional	NA	
**Anticholinergic drugs**												
1 Trihexyphenidyl	Clonazepam;Promethazine Hydrochloride;Quetiapine Fumarate;Flunitrazepam;Fluvoxamine Maleate;Risperidone	Maternal Exposure During Pregnancy	Eye movement disorder	Drug Withdrawal Syndrome Neonatal;Hypotonia Neonatal;Irritability;Tremor Neonatal;Fever Neonatal;Somnolence Neonatal	Serious	Other outcomes	Female	2005	0	Healthcare Professional	JP	.
**Gabapentinoids**												
1 Gabapentin	Phenobarbital;Valproate Sodium	Maternal Exposure Timing Unspecified	Congenital eye disorder	Reproductive Tract Hypoplasia, Male;Developmental Delay;Autism Spectrum Disorder;Low Set Ears;Poor Sucking Reflex;Tooth Disorder;Foetal Anticonvulsant Syndrome;Scoliosis;Decreased Interest;Weight Gain Poor;Foetal Exposure During Pregnancy;Motor Developmental Delay;Hypotonia Neonatal;Behaviour Disorder;Dysmorphism;Abnormal Palmar/Plantar Creases;Vomiting;Growth Retardation;Weight Decrease Neonatal;Camptodactyly Congenital;Hypotony Maculopathy;Hypoglycaemia Neonatal;Feeding Disorder;Language Disorder;Abnormal Labour Affecting Foetus;Scaphocephaly;Cognitive Disorder;Fine Motor Skill Dysfunction;Spinal Instability;Psychomotor Retardation;Gastrooesophageal Reflux Disease;Intentional Self-Injury;Enuresis;Micropenis;Somnolence;Asterixis;Brain Stem Auditory Evoked Response Abnormal;Strabismus;Kyphosis;Pyelonephritis;Cytogenetic Abnormality;Anxiety;Dysphagia;Body Mass Index Decreased;Personality Disorder;Hypertelorism;Communication Disorder;Mental Disorder;Hypospadias;Stereotypy;Decreased Appetite;Ear Infection;Muscle Spasticity;Muscle Atrophy;Talipes;Bronchiolitis;Malnutrition;Lordosis;Craniosynostosis;Prognathism;Cerebral Palsy;Sleep Disorder	Serious	Hospitalized;Other Outcomes	Male	2000	5 months	Healthcare Professional	FR	-
2 Gabapentin	Clonazepam;Acetaminophen\Butabarbital\Caffeine;Acetaminophen\Butalbital\Caffeine;Buprenorphine Hydrochloride\Naloxone Hydrochloride;Oxycodone;Diazepam;Ranitidine	Anxiety;Migraine;Pain	Eye disorder	Low Birth Weight Baby;Foetal Hypokinesia;Poor Feeding Infant;Tremor;Infantile Vomiting;Paternal Exposure During Pregnancy;Crying;Neonatal Respiratory Distress Syndrome;Dyspnoea;Sepsis;Foetal Growth Restriction;Foetal Exposure During Pregnancy;Behaviour Disorder;Hyperbilirubinaemia;Bradycardia Foetal;Premature Baby;Drug Withdrawal Syndrome Neonatal;Hearing Disability;Gastrooesophageal Reflux Disease;Exposure Via Breast Milk	Serious	Hospitalized;Other Outcomes	Male	2016	0	Consumer	NA	-

	Hydrochloride;Acetaminophen\Hydrocodone Bitartrate;Alprazolam;Buprenorphine Hydrochloride;Ranitidine;Diazepam;Clonazepam;Morphine Sulfate;Betamethasone;Buprenorphine;Promethazine Hydrochloride											
3 Gabapentin	Natalizumab;Mirtazapine;Dalfampridine;Enoxaparin Sodium	Maternal Exposure Timing Unspecified	Retinopathy	Neonatal Respiratory Distress Syndrome;Foetal Growth Restriction;Selective Eating Disorder;Low Birth Weight Baby;Foetal Exposure During Pregnancy;Hypospadias;Atrial Septal Defect;Osteopenia	Serious	Hospitalized; Other outcomes	2013	NA	0	Healthcare Professional	NA	-
4 Gabapentin		Trigeminal Neuralgia	Hypermetropia;	Congenital AnomalyMaternal Exposure During Pregnancy	Serious	Congenital Anomaly	Female	2003	0	Healthcare Professional	NA	
5 Gabapentin	Carbamazepine;Phenytoin		Vision Abnormal Neonatal	Developmental Delay;Maternal Drugs Affecting Foetus	Serious	Congenital Anomaly	NA	2001	2 years	Healthcare Professional	NA	
**Antipsychotics**												
1 Quetiapine	Venlafaxine Hydrochloride;Propranolol Hydrochloride;Lithium	Product Used For Unknown Indication	Amblyopia	Developmental Delay;Speech Disorder Developmental;Disturbance In Attention; Foetal Exposure During Pregnancy;Facial Paralysis; Drug Interaction	Serious	Congenital Anomaly;Other Outcomes	Male	2018	1 hour	Healthcare Professional	GB	-
2 Quetiapine		Affect Lability; Product Use for Unknown Indication	Optic Discs Blurred	Foetal Exposure During Pregnancy;Nasal Congestion;Hypertonia Neonatal;Diarrhoea; Acoustic Stimulation Tests Abnormal;Sneezing;Agitation Neonatal	Serious	Other Ouctomes	Male	2019	1 day	Healthcare Professional	DE	
3 Quetiapine		Schizophrenia	Photophobia	Haemangioma Congenital;Foetal Exposure During Pregnancy;Disturbance In Attention;Hyperreflexia;Neonatal Behavioural Syndrome; Agitation;Facial Asymmetry	Serious	Congenital Anomaly;Other Outcomes	Male	2013	1 day	Healthcare Professional	DE	
1 Olanzapine	Insulin	Schizophrenia;Type 2 Diabetes Mellitus	Eye Movement Disorder	Hypotonia Neonatal;Atrial Septal Defect;Neonatal Hypoxia;Foetal Exposure During Pregnancy;Ventricular Septal Defect;Cardiac Septal Hypertrophy;Selective Eating Disorder	Serious	Congenital Anomaly;Other Outcomes	Male	2017	1 day	Healthcare Professional	DE	-
2 Olanzapine	Oxazepam	Bipolar Disorder	Nystagmus	Psychomotor Retardation;Astigmatism;Autism Spectrum Disorder;Foetal Exposure During Pregnancy	Serious	Disabled	NA	2013	o	Healthcare Professional	FR	-
3 Olanzapine	Sennosides;Risperidone;Promethazine Hydrochlori	Analgesic Therapy;Insomnia;Product Used For	Visual Impairment	Maternal Drugs Affecting Foetus;Drug Withdrawal Syndrome Neonatal;Foetal Alcohol Syndrome; Dextrocardia	Serious	Congenital Anomaly;Other Outcomes; Disabled	Male	2013	1 year	Healthcare Professional	GB	

	de;Haloperidol;Alcohol;Zopiclone;Chlorpromazine;Nitrous Oxide\Oxygen;Ergonovine\Oxytocin;Cyclizine	Unknown Indication;Schizophrenia;Sleep Disorder										
4 Olanzapine	Paroxetine; Alprazolam; Oxazepam	Product Use for Unknown Disorder	Eye Movement Disorder	Dyskinesia Neonatal;Oxygen Saturation Decreased;Hypotonia Neonatal;Extrapyramidal Disorder;Foetal Exposure During Pregnancy	Serious	Hospitalized;Other Outcomes	Male	2013	0	Healthcare Professional	FR	-
5 Olanzapine		Schizophrenia	Visual Impairment	Maternal Drugs Affecting Foetus; Maternal Exposure During Pregnancy	Serious	Other Outcomes	Female	2009	3 months	Healthcare Professional	FR	-
6 Olanzapine	Venlafaxine		Eyelid Disorder	Head Lag;Maternal Exposure During Pregnancy;Dyskinesia Neonatal;Infantile Apnoea;Floppy Infant;Selective Eating Disorder;Cyanosis Neonatal;Hypotonia Neonatal;Vascular Skin Disorder;Neonatal Disorder	Serious	Hospedalized	Female	2005	1 day	Healthcare Professional	US	-
7 Olanzapine	Sertraline	Bipolar Disorder; Depression	Visual Acuity Reduced;Optic Nerve Hypoplasia; Nystagmus	Maternal Exposure During Pregnancy	Serious	Congenital Anomaly; Disabled	Male	2003	4 months	Consumer	NA	
1 Clozapine	Haloperidol		Eye movement disorder	Poor Sucking Reflex;Muscle Tightness;Dysphagia;Foetal Exposure During Pregnancy;Haemorrhage Subcutaneous;Staring;Scleral Discolouration;Muscle Spasms;Vomiting;Gaze Palsy;Muscle Twitching;Oedema Peripheral;Yellow Skin;Cyanosis;Drug Withdrawal Syndrome Neonatal;Irritability;Nail Discolouration;Oedema	Serious	Hospedallzed	Male	2014	38 days	Healthcare Professional	CN	Zhu Mingzhe. Clozapine and haloperidol-induced neonatal withdrawal syndrome: A case report. Zhonghua Er Ke Za Zhi. 2011;49 (10):1-4
